# In-Hospital Mortality Among Critically Ill Patients With Atrial Fibrillation (AF) Versus Patients Without AF

**DOI:** 10.7759/cureus.18761

**Published:** 2021-10-13

**Authors:** Tooba Mehreen, Wasib Ishtiaq, Ghulam Rasheed, Nusrat Kharadi, Sara S Kiani, Anum Ilyas, Muhammad Ahmed Kaleem, Kiran Abbas

**Affiliations:** 1 Department of Critical Care, Shifa International Hospital Islamabad, Islamabad, PAK; 2 Department of Internal Medicine, Shifa International Hospital Islamabad, Islamabad, PAK; 3 Department of Critical Care Medicine, Tabba Heart Institute, Karachi, PAK; 4 Department of Critical Care Medicine, Shifa International Hospital Islamabad, Islamabad, PAK; 5 Department of Medicine, Shifa International Hospital Islamabad, Islamabad, PAK; 6 Department of Medicine, Shifa Tameer-E-Millat University Shifa College of Medicine, Islamabad, PAK; 7 Department of Medicine, Jinnah Postgraduate Medical Centre, Karachi, PAK

**Keywords:** prognosis, in-hospital mortality, intensive care unit, afib, atrial fibrillation, arrhythmia

## Abstract

Introduction

Atrial fibrillation (AF) is one of the most frequent arrhythmias observed in the intensive care unit (ICU). The present study assessed AF as an independent risk factor for mortality among patients in the ICU setting.

Methodology

A prospective cohort study was conducted at the medical ICU in a tertiary academic medical center from September 2020 to January 2021. All critically ill patients, irrespective of gender, who were admitted for at least two days in the ICU were eligible to partake in the study. Individuals in the cardiovascular surgical ICU and the trauma ICU were not eligible. Demographics, clinical history, the occurrence of AF, fluid input and output, echocardiography, drug history, and hospital mortality were recorded during the first week of admission. Patients were divided into two groups.

Results

Patients with AF had significantly higher in-hospital mortality, 27 (73%), and longer hospital stays (11.61 ± 7.01) as compared to patients who did not suffer from AF (p<0.0001). The mean length of stay in ICU was 10.32 ± 5.92 and the duration of mechanical ventilation was 7.05 ± 6.16 days in the AF group which was significantly higher than patients who did not have AF (p<0.0001). No significant difference was found in mortality rate between new-onset and recurrent AF among the patients; albeit the latter was higher (60% vs 81.8%, p=0.142).

Conclusion

The present study indicated that AF was a predictor of mortality hence, associated with poor patient prognosis. The occurrence of AF was associated with high in-hospital mortality and longer hospital stay. Further large-scale studies should be conducted to explore other socio-demographic and clinical risk factors.

## Introduction

Atrial fibrillation (AF) is one of the most frequent arrhythmias observed in the intensive care unit (ICU) [1]. Some studies suggest that its incidence in non-cardiac ICUs is as high as 25% [2]. Although pre-existing AF is highly prevalent in the elderly population with multiple comorbidities who have numerous risk factors for admission to intensive care; new-onset AF, on the other hand, can be set off due to substantial structural changes and arrhythmia-associated risk factors faced during stages of critical illness. New-onset AF is defined as the presence of AF among patients who had no previous history of AF in contrast to those with a positive history of AF, i.e., recurrent AF. The mechanisms involved might include inflammation and ischemia, fluid overload, electrolyte imbalance, adrenergic overstimulation, and use of vasopressors and inotropes in critical illness, which not only reduce the efficacy of treatments but also lead to early relapses [3].

Moreover, it has been found that physiological changes during sepsis can cause atrial tissue to become more prone to AF, leading to a greater than six times the risk for suffering from AF in contrast with other contributory factors for hospital admission [4,5]. Studies indicate that individuals in sepsis developing AF usually do not carry a history of cardiac morbidity, which is generally associated as the foremost risk of AF, albeit almost around half of the sepsis patients who develop new-onset AF suffer from recurrent AF post-discharge [6].

The sudden loss of systole in the atria along with the rapid ventricular rates during new-onset AF results in reduced cardiac output and acute hemodynamic instability, precipitating acute heart failure, and thromboembolic complications [7]. Earlier, several reports have suggested a high rate of sudden deaths and increased hospital mortality linked with the occurrence of AF in the stages of critical illness [5,7-10]. Lately, an interesting study by Chen et al. has highlighted new-onset AF as an independent risk factor of two-months ICU mortality in patients admitted in the medical ICU [11]. An article published by Shaver et al. reported that AF occurred in 13% of patients, 7% of whom had no prior AF with new-onset AF being linked with raised diastolic dysfunction and use of vasopressor [10]. Furthermore, an increased positive fluid balance was also linked to new-onset AF [2]. Recently, French and EuRopean Outcome Registry in Intensive Care Unit (FROG-ICU), a large multicenter study reported that during ICU stay, new-onset AF had a greater in-hospital mortality rate in contrast to the no AF group (47% vs 23%, p<0.001) as well as recurrent AF. In their study, new-onset AF showed a greater risk of death after being discharged from the ICU as compared to those without AF (p<0.001) [12].

It is undecisive whether the relation between increased death rate and AF among individuals admitted in the ICU setting is solely due to AF or that AF only indicates the severity of the disease. Data on the significance of the correlation between new-onset or recurrent AF and mortality is limited. Moreover, the impact of fluid balance, use of vasopressors, or other therapeutic protocols for AF on the overall mortality rate still needs to be ascertained. Since the association of new-onset AF in critical illness with mortality is still debated and no such study has been done in our setup, so I suggest this study. Our primary goal was to evaluate whether the occurrence of AF in critically ill patients was associated with greater ICU mortality, irrespective of any underlying medical conditions. Furthermore, the present study assessed whether individuals with new-onset AF present with other contributory factors in contrast to patients with recurrent AF.

## Materials and methods

A prospective cohort study was conducted at the medical ICU in a tertiary academic medical center from September 2020 to January 2021. A simple random sampling technique was employed to recruit participants. All critically ill patients, irrespective of gender, who were admitted for at least two days in the ICU were eligible to partake in the study. Individuals in the cardiovascular surgical ICU and the trauma ICU were not eligible. Demographics, clinical history, the occurrence of atrial fibrillation (AF), fluid input and output, echocardiography, drug history, and hospital mortality were recorded during the first two weeks of admission. A sample size of 100 cases; 50 in each group was calculated with 80% power of the study, 5% significance level, and percentage of in-hospital mortality, i.e., 47% in patients with AF and 23% in patients without AF in critically ill ICU admitted cases.

On recruitment, all patient records were extracted from the database including a history of AF, cardiac disease history, drugs, etc. Evidence of multiple organ failure was also documented in a pre-defined pro forma. Patients were divided into two groups. All medications administered for AF were documented regularly and grouped as rate or rhythm-controlling agents. Patients who do not have previous AF were assessed daily for new-onset AF. Finally, the patient outcome was also observed and recorded including in-hospital mortality, mechanical ventilation duration, length of ICU stay, and length of hospitalization. 

All statistical analysis was performed with SPSS version 22. Numerical variables like age, duration of admission to ICU, were presented as mean and standard deviation. Categorical variables like gender, presence of congestive heart failure, comorbidities, sequential organ failure assessment (SOFA) and acute physiology and chronic health evaluation (APACHE II) scores, sepsis, and in-hospital mortality, were presented as frequency and percentage. For categorical data, univariate analysis using the Chi-square test was performed. P values of ≤0.05 were considered significant. Logistic regression will be applied and models will be built. In the final model, only significant risk factors were included.

## Results

Table 1 demonstrates the association between patient demographics with atrial fibrillation (AF). In a total of 101 patients, 37 patients had AF and 64 patients did not suffer from AF. The median age of the patients with AF was 63.76 ± 16.99 years, whereas the median age in patients not having AF was 51.78 ± 16.67 years (p<0.001). As compared to the patients who were not suffering from AF, patients with AF were older and more likely to have a positive history of hypertension, heart failure, and hyperlipidemia. Patients who did not suffer from AF had few organ failures (p<0.001), more cases of sepsis and shock as compared to those with AF (p<0.03). Incidence of AF was linked with high hospital mortality and longer hospital length of stay (p<0.0001) as compared to those not suffering from AF (p<0.0001). ICU length of stay was also higher along with the duration of ventilation for the AF group (p<0.0001). Most current smokers did not have AF (p<0.068). Patients who had diabetes mellitus type 2, were on dialysis, had a history of angina, congestive heart failure, and cerebrovascular accidents were more prone to developing AF.

**Table 1:** Patient socio-demographic and clinical characteristics and its association with atrial fibrillation. AF: atrial fibrillation, ICU: intensive care unit, APACHE II: acute physiology and chronic health evaluation.

Characteristic	Any AF (n=37)	No AF (n=64)	P-value
Demographics			
Age (years)	63.76 ± 16.99	51.78 ± 16.67	0.001
Male	22 (59.5%)	33 (51.6%)	0.443
Cardiac risk factors			
Current smoker	22 (59.5%)	26 (40.6%)	0.068
Weight (kg)	67.54 ± 11.29	65.56 ± 10.44	0.386
Dialysis	20 (54.1%)	13 (20.3%)	<0.0001
Diabetes mellitus type 2	29 (78.4%)	23 (35.9%)	<0.0001
Angina	30 (81.1%)	20 (31.3%)	<0.0001
Congestive heart failure	18 (48.6%)	6 (9.4%)	<0.0001
Cerebrovascular accidents	14 (37.8%)	11 (17.2%)	0.021
Hypertension	27 (73%)	28 (43.8%)	0.004
Hyperlipidemia	14 (37.8%)	19 (29.7%)	0.4
ICU risk factors			
APACHE II	36.78 ± 14.00	16.78 ± 11.18	<0.0001
Sepsis	30 (81.1%)	33 (51.6%)	0.003
Total number of organ failures	4.11 ± 0.875	2.27 ± 0.877	<0.0001
Shock	30 (81.1%)	33 (51.6%)	0.003
ICU length of stay (days)	10.32 ± 5.92	6.03 ± 4.68	<0.0001
Duration of mechanical ventilation (days)	7.05 ± 6.16	1.22 ± 3.78	<0.0001
Hospital length of stay (days)	11.61 ± 7.01	8.89 ± 5.84	<0.0001
Hospital mortality (n, %)	27 (73%)	2 (3.1%)	<0.0001

Table 2 demonstrates the association of patient characteristics with new-onset versus recurrent AF. AF occurred in 37 patients. Of these, 22 patients had no prior AF (new-onset AF), while the remaining 15 had recurrent AF. Recurrent AF was seen with a greater number of cardiovascular risk factors such as congestive heart failure [14 (93.3%)]; (p<0.0001) and cerebrovascular accidents [nine (60%)]; (p<0.022).

**Table 2:** Association of patient characteristics with new-onset versus recurrent atrial fibrillation. AF: atrial fibrillation, ICU: intensive care unit, APACHE II: acute physiology and chronic health evaluation.

Characteristic	New-onset AF (n=22)	Recurrent AF (n=15)	P-value
Demographics			
Age (years)	60.45 ± 17.55	68.6 ± 15.43	0.146
Male	14 (63.6%)	8 (53.3%)	0.531
Cardiac risk factors			
Current smoker	13 (59.1%)	9 (60%)	0.956
Weight (kg)	69.14 ± 12.75	65.2 ± 8.61	0.27
Dialysis	11 (50%)	9 (60%)	0.549
Diabetes mellitus type 2	16 (72.7%)	13 (86.7%)	0.312
Angina	17 (77.3%)	13 (86.7%)	0.474
Congestive heart failure	4 (18.2%)	14 (93.3%)	<0.0001
Cerebrovascular accident	5 (22.7%)	9 (60%)	0.022
Hypertension	15 (68.3%)	12 (80%)	0.427
Hyperlipidemia	7 (31.8%)	7 (46.7%)	0.361
ICU risk factors			
APACHE II	33.68 ± 14.06	41.33 ± 13.06	0.1
Any sepsis	16 (72.7%)	14 (93.3%)	0.116
Total number of organ failures	4 ± 0.926	4.27 ± 0.799	0.357
Shock	16 (72.7%)	14 (93.3%)	0.116
ICU length of stay (days)	10.95 ± 6.42	9.4 ± 5.17	0.422
Duration of mechanical ventilation (days)	7.64 ± 7.16	6.2 ± 4.41	0.456
Hospital length of stay (days)	11.73 ± 6.07	11.43 ± 8.53	0.91
Hospital mortality (n, %)	18 (81.8%)	9 (60%)	0.142

## Discussion

The aim of our study was to identify whether atrial fibrillation (AF) was an important risk factor for mortality. In our study, those with recurrent AF had a higher rate of mortality (81%) as compared to those with new-onset AF (60%). However, the statistical difference between the mortality rate was insignificant. The mortality rate was high in patients with AF 27 (73%) as compared to those who were not suffering from AF (p<0.0001) during their stay in the ICU.

A study similar to ours was conducted by Arrigo et al. in which new-onset AF was seen with a higher mortality rate and in-hospital death as compared to patients who did not have AF [12]. Patients with new-onset AF were seen with a high rate of post-ICU death as compared to those who were not diagnosed with AF. Yoshida et al. found new-onset AF patients to have a mortality of 26%, and the rate of stroke with patients still admitted in the hospital was 4.5% [13]. The authors also concluded that even though the percentage of patients presenting with AF decreased over time with different treatments, the risk of mortality was still very high. Similarly, Moss et al. found that new-onset AF was linked to high hospital mortality and with a longer stay in the ICU, and no association was found between survival rate after hospital discharge and new-onset AF [14].

Fernando et al. found no significant association between increased mortality in hospitals and new-onset AF in patients who were critically ill [15]. They did, however, find an association in patients who were suspected to have sepsis, shock, and infection. The factors predicting hospital mortality in patients with new-onset AF were age, previous history of congestive heart failure, the severity of the illness, and prolonged AF. Similarly, in our study, recurrent AF was seen with cardiovascular risk factors such as congestive heart failure and cerebrovascular accidents. Karamchandani et al. revealed that new-onset AF was more frequent in critically ill patients and was associated with increased mortality [16]. The authors concluded that a history of vascular disease in new-onset AF patients increases the mortality rate even further. In a similar study, Wu et al. stated that ICU stays and mortality are longer in patients with new-onset AF as compared to patients who did not have AF [17]. Same as previous studies, the authors found risk factors to be age, sepsis, and shock along with organ failures, patients having a pulmonary catheter, fluid loading, and mechanical ventilation. In other studies, new-onset AF was seen with worse outcomes in patients who were critically ill and these patients were also seen with a high incidence of new-onset AF [18,19]. Our study was not without limitations. For instance, due to a smaller undiversified patient subset, the study findings could not be generalized to a larger population.

## Conclusions

The present study indicated that AF was a predictor of mortality hence, associated with poor patient prognosis. The occurrence of atrial fibrillation was associated with high in-hospital mortality and longer hospital stay. Further large-scale studies should be conducted to explore other socio-demographic and clinical risk factors.
